# Lectures on the More Important Eruptive Fevers, Hemorrhages, and Dropsies, and on Gout and Rheumatism, Delivered in the University of Pennsylvania

**Published:** 1846-04

**Authors:** 


### Art. III.

*Lectures on the more important Eruptive Fevers, Hemorrhages, and Dropsies, and on Gout and Rheumatism, delivered in the University of Pennsylvania.* By N. Chapman, m.d., Professor of the Theory and Practice of Medicine, &c. &c.—*Philadelphia,* 1844. 8vo, pp. 448.

The present volume is precisely of the character of the former one, by the same author, reviewed in our Thirty-seventh Number. It contains much good matter of various kinds, the results of great experience and reading, and many sound practical observations; but the whole is enounced amid such confusion of style and arrangement, that the task of perusal, and even of comprehension, becomes irksome and difficult. But we shall not dwell on these defects, but proceed, as on the former occasion, to glean from the heterogeneous mass such things as may be most interesting and useful to our readers.

Smallpox. Dr. Chapman writes with some authoritativeness of the comparatively modern origin of this disease. "From the historical evidence," he observes, "it results that the disease, probably, was developed at the siege of Mecca, for the first time, though under what peculiar circumstances generated we have no real information." But if we allow that the disease appeared from time to time during the three centuries which elapsed between the time of Mahomet and Rhazes, although no clear description of the phenomena which it presented has come down to us, it surely requires no stretch of credulity to suppose that undescribed visitations of the disease may have existed during many centuries previous to the birth of Mahomet. Ozanam, no mean authority, considers the description of Aëtius sufficiently characteristic of the disease; and whatever diversity of opinion may exist regarding the time when Aëtius flourished, no one will place it later than the fifth century. The description by Eusebius of a febrile distemper, as occurring A. D. 311, rendering thousands of women and children blind,—the reference of Hippocrates to some fevers which are pustular and dreadful to behold, *οἱ δὲ πεμφιγώδεες ἰδεῖν δεινοί*,—and the tradition of various eastern nations regarding the ambiguity of the disease, independent of allusions in various classical authors to some at least similar affection,—give plausibility to the opinion, that a wish to connect the ideas of moral and physical evil, rather than a studious regard to accuracy, led authors to fix the origin of the disease on the era of Mahomet, and authorise us in discountenancing all dogmatical attempts to settle a question involved in obscurity, which no illumination of modern learning can be expected to dissipate.

Of the circumstances attending the rise and spread of smallpox, Dr. Chapman, from his own experience, writes as follows:

"In 1823, when smallpox appeared in Philadelphia, after a long interval, it could not be traced to any imported or derivative source of contagion. Cases sprung up, as it were, spontaneously, at a distance from each other, independent of any probable intercourse, wearing universally a most formidable character, and the failures of variolation, and especially of vaccination, were numerous, with some few examples of the disease previously had in the natural way, affording no security. As further proof of the dominant epidemic influence at the time, it may be said, that in the whole compass of our experience, never was exhibited such a tendency to cutaneous affections—every disease, whatever might be its nature, displaying in its course some eruptive appearance, and often of the most anomalous character and aspect." (pp. 25-6.)

Notwithstanding the plausibility of the conjecture which such facts countenance, that the contagion of smallpox is sometimes generated anew, our author is not disposed to adopt it.

"To me," he observes, "it seems probable that the semina of contagion, like those of plants, or ova of animals, and especially of insects, may remain dormant for an indefinite period. As the latter are hatched into existence by a proper degree of temperature and other propitious circumstances, so it is required, to bring the former into activity, a peculiar constitution of atmosphere. We are not wanting in proof that the seminal principle, in each of the instances cited, will endure for a long term of years in a latent state, waiting, as it were, for the vivifying impulse to be supplied: and may it not be equally true in regard to contagion? The musquito, the locust, not to enumerate more examples, disappear for a protracted season, having deposited their eggs, to be awakened into life at some favorable conjuncture. Every agriculturist is aware of the reversions of certain plants, at remote and irregular periods, the seeds of which must have remained in the soil. As clearly does it seem, that the material of this, and all other contagious diseases, is governed by a similar law. Much reliance, I am aware, has been placed on the doctrine of equivocal generation, in the explanation of some of the preceding phenomena; but can it be reasonably credited, that any fortuitous combination of elements, which this doctrine supposes, is productive of such definite results?" (p. 27.)

We cannot avoid remarking that some of the analogies noticed in the above paragraph possibly involve truths calculated to illustrate the phenomena of epidemic diseases. Among all the hypotheses yet constructed to explain the origin of some of the most remarkable epidemics, we believe it may be said that the animalcular is the only one capable of being reconciled with the singular phenomena which attend the visitation of these diseases. Dr. Chapman concurs with Heberden and Haygarth in the opinion that the contagious property of variola does not occur till after the appearance, and perhaps maturation, of the eruption; but thinks it probable that, as in the instance of the vaccine affection, the contagion may exist in the vesicle as well as the pustule. He differs from those who believe the poisonous effluvia may escape from the lungs as well as the skin, thinking it evident, that, as the lungs are destitute of the eruption, they cannot concur in the generation or emission of the contagious halitus.

The insusceptibility of some individuals, and even families, to the contagion, is worthy of notice:

"Foderé mentions the very remarkable instances of those of both of his grandfathers having escaped, and that he himself, though in advanced age, and often subjected to its contagion, had never had the disease. It has, indeed, been calculated, that one in fifty has such a constitutional immunity. But this estimate seems too large, and in no instance is an exemption from it to be confidently relied on. Examples are numerous of persons who, after escaping for a term of years, where affectability being awakened by some mysterious change of condition, the disease attacked, and for the most part, fatally." (pp. 24-5.)

His suggestions on the treatment of smallpox do not claim any particular comment. He justly observes, that it must be adapted to the condition, whether inflammatory, congestive, or mixed. He attaches considerable importance to the use of emetics where not distinctly contraindicated in variola, as in all eruptive fevers, and relies mainly on laxatives and a cool temperature, employing bleeding or cupping when the fever is of an inflammatory character. We differ from the professor as respects his recommendation of the use of diaphoretics, such as antimony and acetate of ammonia, remedies which we consider calculated to increase the quantity of eruption. It is an interesting fact, that the cooling practice in smallpox was distinctly recommended by Rhazes, but was superseded by the dogmas of a false pathology. John of Gaddesden recommended the patient to be wrapped up in red cloth. Sennertus, in the fourteenth century, urged the importance of a warm chamber, and Diemerbroeck advised his patients to use the thickest blankets, and avoid the risk of change of linen. It was reserved for the sagacity of Sydenham to rectify the error, and dictate a return to the ancient practice. Dr. Chapman, considering that, especially in the confluent state, the condition of the skin resembles that of a burn, occasioning nervous inquietude and death, in a similar manner, suggests the application of flaxseed mucilage, and in the asthenic condition, camphorated or Kentish ointment.

Our veteran author dwells on the importance of preventing the marks which are so detrimental to female beauty. After enumerating the numerous suggestions and practices noticed in books, as mercurial ointment, camphor ointment, calomel ointment, a corrosive sublimate wash, covering the face with gold leaf, puncture of the pustules, &c., he adds:

"My own practice has been to subdue inflammation as far as possible by cooling lotions, to open the pustules as soon as they fill, and wash them with milk and water, taking care also as far as posssible to keep the face covered. It appears, however, that the most effectual means is the exclusion of light. Experiments made some years ago, at New Orleans, if they can be relied on, and I know of no reason why they should be distrusted, are very satisfactory on this point.

To try the effect of this expedient, a certain number of patients, during the eruptive and maturative stages of the disease, were confined in a dark ward of an hospital, and not a pit or scar, or other deformity of the skin, was left, though some of them had the disease most violently, even in the confluent form. These experiments were originally performed by Dr. Picton, a graduate of the University of Pennsylvania, and were contained in his inaugural thesis, which I had published on account of its merits. Notices of their confirmation I have lately seen in the medical journals of several of the European countries." (pp. 43-4.)

Medicus, Meige, Professor Schroider of Gottingen, Odier, of Geneva, Schevanke, and some other respectable authors, are of opinion that inoculated smallpox is not contagious.

"'To this question,' says Walkinson, 'I have paid particular attention since the establishment of a dispensary for general inoculation, and can with truth affirm that not a single instance has occurred in that charity in which the contagion was spread by an inoculated patient. Where the chance of spreading it has been apparently great, I have been very strict in my inquiries.' He adds, 'that some inhabited narrow streets, or little courts, and ground floors, the doors of which were kept open, and though surrounded by persons obnoxious to the disease, and especially by a set of children, who continually played before the houses, a few yards only from the sick, all escaped infection.' Exceedingly strong is the testimony of Mr. Holwell, at least in regard to India. Living in that region for thirty years, and during the period inoculating multitudes, he affirms positively that it never spread the infection, as is commonly imagined in Europe." (pp. 45-6.)

Dr. Chapman observes that this opinion is supported by various analogies, especially by that of the vaccine affection, which,

"While contagious, is totally void of infection, and as it is probably modified smallpox, it may be rationally conceived, that by inoculation such a change is wrought in the latter disease as to bring it into the same category." (p. 46.)

The professor claims for his own country much credit in the establishment of the practice of inoculation.

"In 1721, when it had only been adopted in England in a few instances, and these chiefly malefactors, whose punishment by death was commuted, and a load of prejudice still existed against it, Boylston of Boston, a name deservedly high in the annals of American medicine, against the unanimous opinion of the other physicians of the city, and in direct contravention of an edict of the municipal authorities, carrying with it heavy penal consequences, had the intrepidity to inoculate two hundred and eighty-six persons, of whom six only died. The disease was prevailing extensively at the time, and out of 5759 persons, who took it in the natural way, 884 perished.

"To escape from the conviction of the inestimable advantage of the process was impossible after so successful an experiment, and when the result transpired in England, doubt and hesitatation were rapidly removed, and the practice in no long time came to be generally adopted." (p. 49.)

The reader will admire the very natural complacency of our author, who concludes his review of the obstacles opposed to the spread of inoculation in many other countries with this remark:

"Enough perhaps has been said to show that this in common with every other improvement, wheresoever it may emanate, was eagerly received, and skilfully pursued by our own, in the proper sense of the term, truly enlightened country, whose mind has never been prevented by vulgar prejudice, or weakened by fearful superstition, or its determinations thwarted by aristocratic influence, or the power of antiquated corporations, or that of government itself, so perniciously felt in the old world." (p. 51.)

Cowpox. It is unnecessary to follow the professor in his references to the facts tending to establish the identity of variola and vaccinia, as the readers of this journal are familiar with the admirable and perfectly conclusive experiments of Mr. Ceeley on this subject.

In America it is thought convenient to employ the scab instead of the pellucid fluid, and to this cause Dr. Chapman thinks it not improbable that the alleged greater frequency of failure in the prophylactic powers of vaccination may be attributed.

The visitation of smallpox which commenced in Scotland in the winter of 1818, and prevailed nearly contemporaneously in England, especially in Norwich, and on the Continent, after progressively spreading through Europe, crossed the Atlantic, diffused itself over Mexico, South America, and the Antilles, and passing to the East Indies, pervaded nearly the whole world, proving one of the most extensive epidemics on record. In July 1823, four cases of strongly marked variola occurred at the same time, in widely separated parts of Philadelphia, in individuals who had not had any intercourse with one another. Such cases multiplied. The disease as it extended presented every variety from the mildest varicella to the most malignant smallpox. The former variety, however, was the most prevalent.

We quote the author's account of some peculiarities of this visitation.

"The protective powers of vaccination proved with us infinitely less than elsewhere. From data tolerably authenticated it is computed that between 4000 and 5000 failures of this process took place, and I have not been able to collect more than 30 instances of alleged secondary smallpox, and very few were the previous attacks in the natural way, or so violent by inoculation as to have left any marks behind.

"Curious is the fact that neither Dr. Physick nor myself, on this or any other occasion, ever met with an unequivocal instance of secondary smallpox. Many of the cases reported to be such I visited, and detected a source of deception which ought to be guarded against in the investigation of the subject. The disease chiefly prevails among the most stupid and ignorant classes of society, by whom the term inoculation is only employed, and hence they are apt to report themselves as having been variolated, when really the act was that of vaccination.

"The following table, taken from the report of Drs. Mitchell and Bell, who had charge of the smallpox hospital, is interesting in several views. It furnishes a statement of the results of 148 cases of the disease. There were 47 cases in persons who had been previously affected by vaccination, none of which died. Eight cases occurred in persons previously affected with smallpox, of whom 4 died and 4 recovered. Ninety-three cases where in persons who had not had either disease before, of which 52 died, and 41 recovered."

"Of the whole number, 69 were whites, and 79 persons of colour. Two out of the 8 persons who had suffered from smallpox a second time, took it the first time naturally or without inoculation. Eight of those vaccinated were so during the prevalence of the epidemic, and some of the mildest cases were in the persons of those who had been vaccinated upwards of 20 years before.

"The disease, in conformity with most others dependent on a specific contagion, gradually declined on the accession of warm weather, and by the 1st of June entirely subsided. Next winter, however, it reverted, though sparingly, and thence ceased, with perhaps here and there a separate case, till the succeeding winter, when it again returned very much in the same manner as before. From 1825 to 1827, so little was seen of it that hopes were entertained of its disappearance, when it once more revisited our city to a considerable extent. During the next two years, it became nearly extinct, and so continued till the winter of 1830, on which occasion numerous cases occurred. Not much was heard of it after that period, though occasionally solitary instances were met with. But in 1833 it again revived, and spread widely; since which, with the exception of the year 1840, we have had scarcely any of it, and probably the epidemic has become exhausted. Each of its renewals has been marked by nearly the same phenomena, varied chiefly by gradations of violence, and in every instance preceded by varicella, scarlatina, rubeola, as well as by an infinity of other cutaneous affections. It is not to be supposed that we were exclusively the victims of this disease; nearly all our cities, and many portions of the country, have been exposed to its ravages during the same period, though probably not in the same degree." (pp. 90-2.)

Dr. Chapman advocates the opinion that a disposition to smallpox, although interrupted by vaccination, is apt to be reacquired at a distant interval, and thinks it not improbable that a recurrence to variolation may in some instances be found an expedient measure.

We are concerned that a physician of so much experience should lend the force of his reputation to disturb the confidence of the public in the efficacy of vaccination, notwithstanding the evidence which may be collected, even from his own incidental statements, that a vaccinated individual incurs less risk of death from smallpox than one who has already passed through an attack of that disease. We will mention a few facts, which cannot be gainsayed, in proof of our assertion.

In the Royal Military Asylum at Chelsea, between 1803 and 1833, 26 cases of secondary smallpox appeared, of which 3 died; whereas only 24 cases of variola occurred after vaccination, without a single death. In the Smallpox Hospital of London, in 789 cases of smallpox after vaccination 46 died; but of 9 cases of secondary smallpox, 2 died. But the observations of M. Bousquet, during an epidemic at Marseilles, establish the same truth from the magnitude of the scale more forcibly. In a population of 40,000, of whom 2000 had previously suffered from smallpox, 200 had a second attack; 4 died. Among 30,000 individuals who had been vaccinated, 2000 contracted smallpox, and 20 died; thus, 1 in 500 was the proportion of deaths in those who had passed once through that disease; but among the vaccinated, only 1 in 1500.

Indisputable evidence of the efficacy of vaccination is supplied by the records of the army, the precautions employed to secure the certainty of vaccination having almost excluded smallpox from the military reports. This single fact affords conclusive evidence that the supposed inefficacy of vaccination must be attributable to its omission, or imperfect performance. If time renewed the susceptibility, the majority of cases of smallpox after vaccination should occur in adults, but the reverse is remarkably can credit the statement of Dr. Percival of Dublin, that the disease was the case. From the registrar-general's reports it appears that in the year 1838, in England, of 8706 who died of smallpox, 7575 were under the age of five. In Ireland, during the year 1841, of 58,006 cases, 49,038 were under the age of five. The simple explanation of the prevalence of smallpox is doubtless the neglect of vaccination.

In France, the attention of the legislature has increased the proportion of the vaccinated from one half to five eighths, and the annual proportion of deaths from smallpox has diminished in a remarkable degree.

Measles. A visitation of measles has been thought to occur every seven years. Professor Caldwell affirms that "beginning in 1772, and passing down to a period including fifty years, it prevailed epidemically in this city and vicinity every sixth year." With the exception of influenza, it pervades a country more rapidly than any other epidemic. In 1821, it overran in a few months nearly the whole of the United States.

The experiments of Home, confirmed by Wachsel, appeared to show that measles could be communicated by inoculation. The accuracy of these experiments was questioned by Cazenave. Dr. Chapman states that similar experiments with the blood, mucus, and tears were performed at the Philadelphia Dispensary without success; and he is disposed to attribute the occurrence of measles in the numerous cases in which Von Katona of Hungary inoculated with the fluid of the vesicles mixed with tears, to incidental exposure to infection. We cannot be satisfied with this explanation; the number of the cases treated by Katona exceeding 1000, the eruption occurring uniformly about ten days after inoculation, and the disease proving mild.

We agree with Dr. Chapman in assigning from ten to fourteen days as the incubative period of measles.

Scarlatina. The chapter on scarlatina contains several statements worthy of quotation. Sir Gilbert Blane never saw an individual except one affected with scarlatina above the age of forty. Dr. Chapman attended a patient suffering from the disease at the age of eighty, but he never knew a very young infant take it, however much exposed. Reil witnessed an epidemic in which the disease was almost confined to patients between the ages of fifteen and twenty-five. Different visitations of scarlet fever afford a remarkable contrast in the degree of severity. During the epidemic at Paris in 1743, scarcely an individual who was attacked recovered; on the contrary, various visitations related by Morton and Sydenham were peculiarly mild.

"It is," writes Dr. Chapman, "one of those diseases that very conspicuously appears to have its cycles, in which the most opposite character is presented. The whole history of it warrants this conclusion. It has so prevailed from the earliest settlement of our country. Nearly half a century ago I recollect it as the terror of our community, and so it continued for some time; but, from about 1801 to 1830, it recurred at certain intervals, uniformly with such extraordinary benignity, that it rarely gave any trouble or anxiety in the management. I have heard the late Professor Physic declare that, during that lengthened period, he did not lose a patient in it. For the last ten years, however, it has proved very much the reverse, some seasons highly malignant and frightfully fatal as well immediately among us as elsewhere." (p. 138.)

The contagion of scarlatina adheres to fomites with much tenacity. We introduced into that city by a box of toys from London, which had been exposed to the contagion.

"Dr. Elliotson mentions that for nearly two years all the children admitted into a particular ward under his care were seized with scarlatina, in consequence of a patient with the disease having been in the ward at that remote period, and this in despite of white-washings and other cleansings." (p. 134.)

At the same time, we attach greater importance to the power of free ventilation in dissipating the contagion, and place no reliance on the famous story of Hildebrand, who assures us that "as soon as he arrived in Padolia, scarlatina broke out and spread most widely, which he ascribes to the retention of contagion in a coat he had worn in the disease a year previously in Vienna." (p. 134.)

In this, as in many other parts of the volume, we have to regret that the writer should associate statements of every variety of probability, without attempting to define the principles which are to guide our credulity, and regulate the surrender or refusal of our assent.

Our author has evidently paid much attention to the duration of the incubative periods of contagion :

"That of scarlatina," he observes, "is said to be from five to six days. My own conviction is that it is usually greater, though I speak diffidently on the point, having been unable to satisfy myself in regard to it. Equally subjected apparently to infection, I have seen individuals break out with the disease from the third to the eleventh day; and we have had some cases lately reported, where, in a family of eight, the interval varied from the seventeenth to the twenty-sixth day, the average being seventeen days." (p. 135.)

Dr. Chapman's views on the treatment of scarlatina are for the most part judicious. After noticing the contrariety of opinion among books and practitioners regarding bleeding, a measure supported by Morton and Armstrong, but opposed by Sims, Withering, Clarke, and Willan, he observes :

"This contrariety of sentiment in relation to the remedy, I presume must be referred to its having been applied under opposite circumstances of the disease, and of course attended by very different results. Directed with discrimination, it cannot fail, according to my experience, to be beneficial, and often even indispensable. Convinced of this, my own practice, indeed, and which is that of our physicians generally, is to bleed in nearly every case of high and active excitement Yet it is true that the loss of blood has no direct curative tendency in the disease, it only abating action, without changing or subverting it, and as usually not well borne to any great extent ; it is not safe to detract it with the same freedom as in the more purely phlegmasial affections, or perhaps to the amount that the existing indications in the case itself would seem to demand. Collapse, frightful and sometimes even fatal, I have repeatedly seen to result from an abuse of the practice, and it is always hazardous at an advanced stage of the disease. Local affections merely may be removed by leeches or cups." (pp. 142-3.)

Having observed some striking instances of "the deepest form of collapse" in Asiatic cholera, after resisting all stimulating and arousing means, readily "reacted" by frictions with cakes of ice to the whole cutaneous surface, and the freest consumption of the article internally, Dr. Chapman is inclined to think that similar applications might be useful in the passively congestive variety of scarlatina.

Whenever the disease is excited by an exposure to cold, it is prone to present the malignant form, and is very difficult of cure. The author prefers burnt alum to nitrate of silver as a local application, both to sloughs and membranous exudation on the fauces. He questions, and most justly, the advantage of mercurials in the typhoid variety of the disease, and makes no mention of what many believe to be one of the most valuable remedies in this condition—we mean the chlorate of potash. We concur with him in a favorable opinion of digitalis in many cases of scarlatinal dropsy.

The professor is inclined to allow some weight to various powerful evidence in favour of belladonna as a prophylactic ; from ten to fifteen drops being given night and morning of a solution of two grains of the extract in an ounce of water. *Post hoc ergo propter hoc.*

"Yet (he pithily adds) by the assurance of Hahnemann, the author of the homœopathic doctrines, which, originating in fraud and imposture, and continued by the most atrocious wickedness, that he was equally successful in the infinitesimal dose of a few drops, daily, of a solution which contained no more than the twenty millionth part of a grain of the extract, distrust and even ridicule are cast over the whole affair, and our faith must be withdrawn and suspended till fresh and better evidence is afforded." (p. 151.)

HEMORRHAGE. The work contains a somewhat tedious chapter on hemorrhage, in which the author takes great pains to establish an important truth, distinctly propounded by the best writers, but not yet sufficiently recognized by the public, namely, that "vital" hemorrhage is an effusion from the exhalents, not a result of rupture of vessels. He judiciously remarks :

"Too much importance is by many attached, in the management of vital hemorrhage, to its suppression. Great alarm is created by it in the individual himself, as well as in his friends, and from which the medical attendant is not always entirely exempt. Every exertion is therefore made to check it, and this being accomplished, the anxiety which previously existed heedlessly subsides. Lulled into false security, the patient is too often permitted to revert to his former habits, without any permanent plan of treatment, till again awakened to a sense of danger by a repetition of an attack, and in this way he proceeds till the complaint is often irremediably fixed. Now, the hemorrhage in itself is comparatively of little moment—for the most part, indeed, beneficial—and the real object of attention should be the correction of the condition giving rise to it, and which, by neglect, in numerous instances leads to the most disastrous consequences." (p. 171.)

In proof of the extent to which exhalation may proceed, we extract a case which Dr. Chapman attended with Dr. Dewees:

"A young man, otherwise in good health, who, for three succeeding days, lost about three pints of blood daily from his gums. Both these and his teeth were remarkably sound to all appearance. By wiping the gums clean, the blood was seen in a moment oozing out from numerous pores." (p. 164.)

*Hemoptysis.* As respects the liability of particular classes to hemoptysis, we are assured by the late Professor Rush, "that those religious denominations who do not sing, and mostly worship silently, are very subject to it, from weakness of lungs, owing to the want in this mode of adequate exercise of these organs ;"

"But," says Dr. Chapman, "my own experience, however, does not confirm this observation. Living in the 'city of friends,' I have seen no peculiar liability in this description of people to be thus affected. Clergymen, on the contrary, are exceedingly subject to hemoptysis, which has been ascribed, though I think erroneously, to the performance of the services of the pulpit. That it cannot be so, to the extent averred, is shown by the comparative immunity of lawyers, and legislators, and lecturers, each of whom harangue more constantly and loudly than preachers. I have, in treating of phthisis, advanced a conjectural explanation of this point. Be it as it may, the fact appears to me unquestionable, that this hemorrhage is far more frequently to be witnessed in connexion with an undue exertion of the lungs than the reverse, or the comparatively quiescent state, to which an allusion has been made." (p. 175.)

Dr. Chapman has observed hemoptysis to occur most frequently during sleep, a circumstance which he attributes to the tendency of a horizontal posture, with bending of the lower extremities, to determine blood to the lungs. Heberden, in a practice of sixty years, never lost a patient from hemoptysis. "This symptom should for the most part carry little further terror than that excited by a suspicion, too often well founded, that it is an outward sign or expression of disease of the lungs, and especially a tubercular state of those organs." In this opinion we concur, believing, with Louis, that, with few exceptions, such as cases arising from plethora, either general or local, *considerable* expectoration of blood is an evidence of the presence of tubercles.

The suggestion to which Dr. Chapman attaches most importance in the treatment is the use of ipecacuanha emetics. "Thirty years' experience of the success of the treatment has inspired great confidence in it." We regret that these thirty years have not conducted him to a more lucid explanation of the efficacy of the practice. "They operate by breaking up the habits and associations which continue the predisposition, and are also well calculated to emulge loaded vessels, and to distribute the blood equally throughout the circulation." (p. 196.)

*Epistaxis.* In the article on epistaxis the learned professor exhibits the aptitude of his memory for rare and wonderful events. Bleeding from the nose has been produced by loud sounds, "as a clap of thunder or the explosion of cannon."

"Blanchard says, he had seen it occur by the ringing of bells. I had a friend at Edinburgh who assured me that he was never exposed to the screechings of the Scotch bagpipes without fulness of the head, often leading to an effusion of blood; and I have somewhere read of an individual in whom the discords of music operated as a sternutatory, beginning with sneezing, and ending in the escape of blood. . . . . . It is sometimes, too, excited by acrid fumes, and may be by pungent or the blandest odours. I once knew an individual who could bring it on by smelling cheese for only a few minutes, and have heard of another in whom rotten apples had a similar effect. Bruyerin, indeed, gives an instance where the soundest apple induced it, and Rhodius tells us that it has followed the smelling of a rose. Moreover, it is occasioned by mental emotions, rage or terror, or a very excited imagination, or intense study, or anxiety with insomnolency." (p. 198.)

Opiates, blisters to the chest, and the internal use of iced water, the author considers valuable auxiliaries in the treatment. Acetate of lead is more effectual in doses of a grain or two at short intervals, than when given in larger quantities.

Epistaxis occasionally assumes an epidemic character. Such an event is related by Morgagni as leading to great mortality in Tuscany and other parts of Italy, and a similar occurrence was observed in Pennsylvania during the year 1823. The quantity of blood lost by epistaxis is sometimes enormous:

"Bartholin mentions a case of forty-eight pounds within a period not given. Rhodius another of eighteen pounds within thirty-six hours; and a respectable writer in the Leipsic Acta Erudita, a third example of not less than seventy-five pounds within ten days. The Ephemera of Natural Curiosities contains a case where the quantity is not stated, from the difficulty of taking an account of it, which continued without cessation for six weeks." (p. 201.)

Dr. Chapman seems to experience peculiar pleasure in recording instances of great losses of blood; he mentions having attended patients affected with intestinal hemorrhage, one of whom lost eight gallons in a fortnight (but recovered); another eight quarts in three days, who died; a third, a pint daily for a fortnight, who ultimately did well; a fourth, three gallons in twenty-four hours. He mentions some other fatal cases under his own care, in which the quantity could not be ascertained, and he refers to the remarkable instance related by Michelotti in the 'Transactions of the Royal Society,' of a young man with enlargement of the spleen, who threw up in two hours more than twelve pounds of blood, and finally recovered. In the chapter on Hemorrhoids similar marvels are related; for example, on the authority of Lieutaud, six quarts in two days; on that of Panaoli, a pint daily for two years; and of Hoffman, twenty pounds in less than twenty-four hours.

*Hematemesis.* Emetics, a favorite remedy with our author, appear, from the narratives which he has given, to have proved very salutary in cases of hematemesis. With this exception he prefers turpentine to every other remedy, administering it after suitable aperients, and in doses of from 20 drops to a drachm, in such complaints.

We do not dispute the occasional usefulness of emetics, which indeed are strongly recommended by Dr. Osborne and other judicious practitioners, but the prepossession of Dr. Chapman in their favour is obviously so strong as to impair the authority of his testimony. One of the cases adduced by him in proof of their value being that of a lady, of whom he never heard after recommending her to "commence a course of emetics." He adds, "It were easy to adduce further cases of a similar kind, though perhaps not so striking."

*Hemorrhoids.* Hemorrhoidal affections are said to be more common in hot, damp, and miasmatic, than in cold and dry countries, and to prevail more in the South than in the North of the United States. They consist, according to our author, most commonly of protruded mucous membrane, as asserted by Colles and Kirby, not often, as alleged by Brodie, of varicose veins. He doubts the accuracy of the popular prejudice against aloes. So do we. As local applications, Dr. Chapman recommends ointments containing lead, laudanum, or mercury, narrow-leaved dock, stramonium, or mullen, the last of which remedies he considers lenitive. When the removal of hemorrhoidal tumours is determined on, he strongly deprecates the use of the ligature, excepting in varicose cases. Referring to Tilanus and others for bad results of this measure, he says, "It is wonderful indeed that such catastrophes have not oftener happened, for no demon of wickedness ever devised and practised greater torture than is here sometimes inflicted." Dr. Physick was accustomed to excise external hemorrhoids, and introduced wire ligatures for those which were internal. Dr. Harris, an American surgeon, "of equal judgment and very wide experience, invariably practices excision." We suspect the practise of Dr. Physick to be the best, and attribute the occasional bad effects of the ligature to its not being applied with sufficient firmness to destroy the vitality of the constricted part.

In a note appended to the chapter on Purpura we find references to some interesting cases of exudation of blood in the manner of perspiration through the pores of the skin ; but none of these are original.

Dropsy. We are inclined to acknowledge the influence of climate in producing a disposition to dropsy, a disease which prevails in chilly and damp regions, but rarely appears in countries such as Egypt, Syria, and Nubia, which are steadily warm and dry. We are not, however, fully satisfied with the opinion apparently countenanced by Dr. Chapman, that a diet of pork or fish conduces to the production of this disease. The following passage contains a curious and characteristic collection of observations materially differing in the degree of probability.

"Many years ago I was called in consultation with the late Professor Physick, to a gentleman from Virginia, with general dropsy, who traced its commencement to his having imprudently plunged into a cold bath, while heated and sweating from severe exercise. We were told by him that the effusion took place a very few hours after coming out of the bath, and that previously his health had been perfect. Nearly about the same time Dr. Physick and myself attended a gentleman from South Carolina, with the same disease, by whom we were informed that, in robust health, returning from a tiresome and dusty ride, he went into a bath, the water of which was so hot that he could scarcely bear it. Continuing in it, however, for only a short time, on leaving it he found his skin very florid, even scalded, and soon experienced the distension of ascites, to which succeeded anasarca, and next hydrothorax, so that when he came under our care he had general dropsy.

"Cases I have seen to follow almost immediately flatulent colic. During the winter of 1812, I attended with the late Professor Wistar, a lad, who having become heated and fatigued by skating, laid [lay] on the ice, and after a short period was seized with colic, attended by a distension of the abdomen amounting to tympanitis. By carminatives, opiates, and external warmth, he was very quickly relieved from pain. But on our next visit a few hours afterwards, we were astonished to find that he laboured under ascites and œdema of the lower extremities.

"In the summer of 1825, a lady from the country consulted me, who after eating water-melon and some other fruits, was attacked, as she said, with colic, quickly converted into tympanitis, and ultimately into dropsy of the abdomen, and of the legs and feet." (p. 265.)

"Draughts of cold water have immediately excited the disease, of which probably the most remarkable instance is afforded by Dr. Haen, who informs us that a large portion of the army of the Emperor Charles V., when proceeding against Tunis, fell into dropsy almost instantly after drinking freely of cold water while heated and exhausted, on a march in hot sultry weather."

"Bateman reports a case still more extraordinary, where the disease was at once induced by fright; and Ludolff de Meza insists on its having occasionally followed violent paroxysms of rage." (p. 266.)

Dr. Chapman has rarely seen a child recover when attacked with peritoneal dropsy, and in this observation he is supported by the testimony of

Dr. Physick. In the treatment of dropsy he speaks enthusiastically of the efficacy of compound jalap powder, "in the dose of a scruple or half a drachm of crem. tart. to 5, 10, or 15 grs. of jalap, with a few drops of ol. carui to prevent griping; so repeated as to keep up almost unremitting discharges from the bowels."

"Exhibited in this manner the results in some cases are promptly effectual and almost astonishing. I have seen in a few days the utmost intumescence and distension entirely removed by this remedy alone. It is therefore with the strongest emphasis, and in the highest tone of confidence that I press it on attention. Never, I can truly declare, have I had more reason to be delighted with any course of practice, in any disease, than occasionally with purging by the combination to which I have alluded." (p. 294.)

His favorite diuretics are an ounce or two of nitrate of potash daily in three pints of sugared water. Two-drachm doses of sweet spirits of nitre, or the following combination: ℞ Tinct. thebaicæ, gtt. x; sp. æth. nitr. ʒij; vini antim. gtt. 40; aq. font. ℥ij.

He suggests the annexed formula as a preeminently valuable hydragogue cathartic: ℞ Cambog. gr. iv; elater. gr. ss; sp. nitr. dulc. ℥j; aq. font. ℥iv. M. A tablespoonful to be taken every two hours.

On the failure of medicines to remove the disease, before resorting to the operation, Dr. Chapman says:

"It will be right to try the effect of a large blister to the abdomen, which I have known in two instances to prove effectual. The first of these occurred in a maiden lady somewhat advanced in life, attended by the late Professor Wistar and myself. Every measure used having failed, and the distension being excessively oppressive, we determined on tapping, the next day. But of her own accord an immense blister was applied in the evening, and at our next visit we were astonished to find the fluid evacuated, it having leaked through the skin of the abdomen to such an extent as completely to have soaked her bed. Exactly such a case is reported by Professor Caldwell, of the Louisville College, and with which I became acquainted from another and equally authentic source." (p. 306.)

*Ovarian dropsy.* In the dissertation on hydrops ovarii, our author expresses his dissent from the doctrine that the disease is owing to hydatids irritating the adjacent surface into effusions, but he considers blows to be a frequent cause. "Lee, no common authority, especially declares that it scarcely admits of doubt, from the progressive enlargement observed of the graafian vesicles, that the cysts supposed to be hydatids often originate in the morbid distension of the former bodies." (p. 311.)

The professor has a great admiration of heroic operations, and falls into the youthful mistake of confounding boldness with skill. He is favorable to ovariotomy, but his own facts are against his doctrine.

*Hydrothorax.* We are happy to find, in a work written by a physician of the transatlantic school, a caution against injudicious bleeding, and we therefore quote with satisfaction the following paragraph:

"Dropsy of the chest not unfrequently follows pertussis, asthma, and protracted catarrh, or bronchitis, especially in weak lymphatic persons. It is also and more frequently consequent on disorganization of the heart and the great vessels, or disorders of the chylopoietic viscera, including the stomach, sometimes misplaced or irregular gout or rheumatism, or repelled or imperfectly cured eruptions; above all scarlatina, as well as a state of anemia, however induced, particularly by excessive losses of blood. The fact is too important not to be mentioned, that it very often happens, where the pleurisy of aged or otherwise feeble people is thus treated, though that disease may be cured, an effusion into one pleura becomes entailed. During my long attendance in the Almshouse Infirmary, such an event so commonly occurred among the inveterate drunkards of that establishment, that I became exceedingly discouraged from the use of venesection to any extent in that affection under these circumstances." (p. 319.)

The Professor mentions, with approbation, a prescription for Hydrothorax, which Dr. Ferriar, after a series of clinical experiments, adopted as the best:—℞ E. elater. gr. j; tinct. scillæ, oxym. scillæ, āā ℥ss; sp. æth. nitr. ℥ij; syr. rhamn. ℥j. The dose, one drachm every three or four hours.

Our readers must not be allowed to lose the narrative of the *walking-off a dropsy*, which will not fail to remind them of the famous story of the Hypochondriac cured by an enforced ride from London to Inverness, to see a certain hypothetical doctor Macdonald.

"The late Professor Rush was in the habit of relating the fact of a poor man, who, despairing of being relieved at home of an inveterate dropsy, determined to seek his advice, and for this purpose travelled on foot several hundred miles. Encumbered by the disease, and exceedingly weakened, he at first could walk only a very short distance. But, as he proceeded, the effusion diminished, and his strength returned, so that he was enabled to complete the arduous undertaking, and presented himself, on his arrival, perfectly recovered. From this anecdote may be deduced the efficacy of exercise in one instance of the disease at least—the value of diffusive professional reputation, and the extent to which it was enjoyed by the great physician from whom it was derived." (p. 375.)

We regret that Dr. G. O. Rees had not an opportunity of examining the blood-corpuscles of this patient before and after his expedition.

*Hydrocephalus.* We find nothing of especial novelty in the article Hydrocephalus. It contains some good practical remarks on the question of depletion in this disease, a subject of great importance, but fully discussed by Dr. Risdon Bennett, in his excellent treatise on that formidable disease.

*Gout.* The following observations on the prevalence of gout in Philadelphia, and its dependence on modes of living, are most valuable. We believe analogous testimony, though perhaps not to the same extent, can be furnished by many of our older British physicians. We are happy to record the doctor's report of the improved habits of his countrymen.

"Of the dependence of gout on the habits of living, no stronger proof can probably be supplied than from the annals of this city. When I commenced my professional career, the disease abounded in the higher circles; and then it was the practice to drink punch in the forenoon, to continue it at dinner, or to resort to ardent or malt liquors, followed by a liberal use of diverse wines, closing the evening with substantial suppers, and stimulating potations. But, in this respect, within the last thirty years, a signal change has taken place. No punch or distilled spirits, and comparatively little malt liquor has been consumed; and the custom of supping is nearly extinct. Temperance has superseded debauchery or excess, and gout, thus deprived of its aliment, is fast perishing away. My opportunities have enabled me to ascertain the fact, that so late as the commencement of the present century, a hundred cases of the disease existed in this community where one is now to be met with, and with few exceptions, these are the remnants of other days serving as memorials of a state of society of which there are scarcely any other traces to be recognized." (p. 385.)

It is remarkable what trivial circumstances will produce gout in predisposed subjects. A cider-manufacturer, with whom Dr. Chapman was acquainted, was obliged to desist from his occupation, finding the mere odour of the liquid sufficient to excite a paroxysm.

The theoretical objections of Sydenham to the use of purgatives in the treatment of gout, are strongly opposed by our author, who places his chief reliance on this class of remedies; but he has sometimes found emetics useful, especially in the case of a gentleman who was constantly attacked with the complaint whenever he visited a badly-drained estate on the banks of the Susquehana.

*Rheumatism.* Dr. Chapman, in reference to the practice introduced by Brocklesby, of administering large doses of nitrate of potash in the treatment of rheumatism, remarks that an ounce of that remedy, daily, is more than most stomachs will bear. This observation is not easily reconciled with his own recommendation of still larger doses in the treatment of dropsy. After noticing atrophy of the muscles as an occasional result of rheumatism, he adds:

"Marasmus of the muscles takes place occasionally without any appreciable rheumatism, or if such attacks as I allude to are of this nature, they must be of the kind vulgarly called dumb rheumatism, devoid of expression by symptoms. The disease mostly comes on with no premonition, sometimes when the individual seems to be in good health, and the first indication of it is the obvious wasting of one or more of the large muscles, usually those of the neck or back, or hips, with corresponding imperfection in the motions dependent on those muscles. Gradually, other muscles become involved to a greater or less extent, and emaciation proceeds in them till it is extreme, and all sorts of distortions and deformities are exhibited. For a long period, the general system seems to sustain little or no detriment from this morbid process, and the digestive functions are, to all appearance, actively performed, but, ultimately, febrile irritation arising, the result is rapidly hastened. Five cases of this extraordinary affection I have seen, all brought to me from the country, the whole of which ended disastrously. Three of these were brothers, and the fourth, a nephew of them, is now under the care of Drs. Jackson, Mitchell, and myself; and I have heard that another member of the same connexion has been similarly affected." (p. 438.)

We are anxious not to overlook any remedies to which a practical physician of so much experience attaches importance. He considers savin, when continued perseveringly till warmth, itching, or eruption, arises, especially useful in lessening rigidity of joints from extravasation, and in arresting absorption in marasmus of the muscles.

In rheumatic affections of the chest, as well as in spasmodic asthma, he has observed much benefit to accrue from administering the juice of the fresh berry of the phytolacca decandria, or an ounce of the tincture three times a day.

Dr. Chapman is not disposed to trouble himself with experiments regarding the virtues of iodide of potassium, for reasons which we will quote:

"Well-tried remedies, like well-tried friends, I always adhere to, and am very slow and reluctant to abandon either without sufficient grounds. To do it in the former instance shows a weak and credulous head, and, in the latter, a capricious and depraved heart." (p. 445.)

But we must abandon our "old friend" Dr. Chapman; for, notwithstanding the merits which we acknowledge him to possess, we are heartily wearied with the confusion of his facts, the intricacy of his thoughts, and the obscurity of his language. The work, as our readers must have perceived, is not deficient in materials which a skilful hand might have erected into a stately monument to the honour of science; but the actual author has suffered these materials to remain a disorderly heap—unshapen and uncemented. We have rarely encountered a more striking evidence of the necessity, to the medical practitioner, of that early mental discipline, without which, the greatest industry, the longest experience, and the most accurate memory, are likely to prove almost useless in the service of science; and we finish our laborious task with increased anxiety, that such improvements in preliminary medical education may speedily be introduced, as shall render infrequent the faults which it becomes our duty so often, and, on the present occasion, so particularly to deplore.

---

### Art. IV.

*Transactions of the Medical and Physical Society of Bombay for the Years* 1842 and 1843. pp. 196; pp. 250.—*Bombay,* 1842-3.

